# Brain Transcriptomics Reveals Molecular Mechanisms of Cave Adaptation in *Triplophysa* Loaches

**DOI:** 10.1002/ece3.72652

**Published:** 2025-12-09

**Authors:** Chunqing Li, Longting Wu, Fang Hu, Shanyuan Chen, Heng Xiao

**Affiliations:** ^1^ School of Ecology and Environmental Science Yunnan University Kunming China; ^2^ School of Life Sciences Yunnan University Kunming China

**Keywords:** adaptive evolution, brain, cavefish, transcriptomics, *Triplophysa*, troglomorphic traits

## Abstract

Understanding the adaptive evolution of brain function in extreme environments remains a central challenge in evolutionary biology. This study investigates the molecular mechanisms underlying cave adaptation by comparing brain transcriptomes of sympatric cave‐dwelling ( *Triplophysa shilinensis* ) and surface‐dwelling (*Triplophysa xiangshuingensis*) loaches (*n* = 5 per ecotype). We generated a comprehensive dataset comprising 60.74 billion clean reads and identified 101,725 Unigenes. Bioinformatics analysis revealed significant differences in brain gene expression between the two ecotypes, with 27,194 differentially expressed genes (12,188 up‐regulated and 15,006 down‐regulated). In *T. shilinensis* , cave adaptation‐associated genes were notably enriched in pathways related to insulin secretion and energy metabolism (*GLUT1*, *IRS1*, *PRKCA/PKCα*, *ACSL*), circadian rhythms and behavior (*CRY*, *FBXL3*, *CLOCK*, *NPY2R*), body coloration (*ADCY9*, *GNQA*), visual development (*RDH8*, *LRAT*, *CNGB1*), and olfactory sensation (*OLFR*, *ADCY3*, *PKA*, *CAMK2*). Strikingly, most differentially expressed genes were down‐regulated in the cave‐dwelling ecotype, a trend further validated by qRT‐PCR. These expression patterns correlate with differential cave adaptation in *Triplophysa*, providing critical insights into the genetic basis of subterranean evolution. Our findings establish a foundational framework for future research on cave acclimatization mechanisms in this genus.

## Graphical abstract

This study compares brain transcriptomes of cave ( *Triplophysa shilinensis* ) and surface‐dwelling (*T. xiangshuingensis*) loaches to uncover molecular adaptations to subterranean life. We identified widespread gene down‐regulation in the cave ecotype, with enriched pathways in energy metabolism, circadian rhythms, sensory perception, and body coloration, revealing key genetic mechanisms of cave adaptation. These findings provide critical insights into the evolution of brain function in extreme environments.

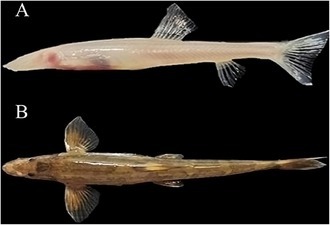

## 1 Introduction

Cave‐dwelling species have evolved a suite of adaptive traits to thrive in perpetual darkness, limited food availability, and elevated carbon dioxide levels—hallmarks of subterranean environments (Aspiras et al. 2015). Among these species, cave fishes exemplify remarkable phenotypic and physiological adaptations, including eye degeneration (Meng et al. 2013), albinism (Li et al. 2020), reduced metabolic rates (Hüppop 1986), disrupted circadian rhythms (Moran et al. 2014), increased fat deposition (Aspiras et al. 2015), insulin resistance, and hyperphagia (Krishnan et al. 2020). Investigating these adaptations not only advances our understanding of evolutionary mechanisms but also sheds light on speciation, functional innovation, and genomic evolution. Cave fishes are characterized by high species diversity, small populations, and restricted distributions, primarily inhabiting karst cave systems and subterranean rivers (Borowsky 2018).

*Triplophysa* belongs to Cypriniformes, Cobitoidea, and Nemacheilidae, and is widely distributed in the Tibetan Plateau and the surrounding areas of Asia. *Triplophysa* exhibits two distinct ecological types: cave‐dwelling and surface‐dwelling. Within the *Triplophysa* genus, both cave‐dwelling and surface‐dwelling species exist, showing significant differentiation. Geographic isolation has led to substantial morphological disparities between cave‐dwelling and surface‐dwelling *Triplophysa* species, impeding gene exchange within this fish group. The cave‐dwelling plateau loaches typically exhibit distinctive adaptations to their underground habitat, including highly regressed or entirely absent eyes, smooth and scale‐free body surfaces, transparent bodies revealing internal organs and subcutaneous blood vessels, elongated pectoral and ventral fins, well‐developed antennae, nasal valves, and enlarged nostrils. These evolutionary modifications in tissues and organs enhance their ability to perceive environmental cues such as food, water flow, and other sensory information in the darkness of cave environments (Trajano 2001). The sympatric distribution of *Triplophysa* fishes is a common phenomenon. Their abundance, small population sizes, concentrated distribution, and pronounced differentiation position them as pivotal members within the realm of Chinese cave fishes, imparting significant research and conservation value.

A notable pair of sympatric *Triplophysa* species is *T. shilinensis* and *T. xiangshuingensis* that are distributed in Shilin County, Yunnan Province, Southwest China. *T. shilinensis* is a cave‐adapted species characterized by complete eye degeneration, well‐developed barbels, and a pointed, elongated body that is scaleless, translucent, and depigmented (Chen et al. 1992). In contrast, *T. xiangshuingensis* possesses small eyes, a large, flattened head, and a pale‐yellow body adorned with distinct dark brown saddle blotches and a fin band (Li 2004).

Regarding the evolutionary history of these two species, there were speculative statements. The ancestors of *T. shilinensis* , which were likely similar to surface‐dwelling species like *T. nanpanjiangensis* , became isolated in subterranean caves through the development of underground river systems in southeastern Yunnan. This geographical isolation drove its troglomorphic evolution, a process linked to the uplift of the Qinghai‐Tibet Plateau and karst formation during the early Miocene radiation of the *Triplophysa* genus. While *T. xiangshuingensis* is also a product of the plateau's uplift and subsequent geological changes, its specific evolutionary pathway remains unclear due to a lack of research data (Chen et al. 1992).

The brain plays an equally vital role in reproductive development, particularly in sexual differentiation and dimorphism. The piscine brain consists of five principal regions: telencephalon, mesencephalon, midbrain, cerebellum, and medulla oblongata. Regional gene expression patterns, especially those related to neurosteroid production and signaling, contribute significantly to observed sexual dimorphism (Liu et al. 2015). For example, (Zhang et al. 2018) demonstrated sex‐specific transcriptional responses in minnows ( *Gobiocypris rarus* ) exposed to tributyltin (TBT), with female brains showing downregulation of circadian genes (*PER*, *CRY*, *REV‐ERB*) while both sexes exhibited upregulated phototransduction genes (GNAT1_2, RH/visual proteins). Such findings highlight fundamental neuroendocrine differences underlying sexual dimorphism in fish.

Recent advances in lipidomics and spatial mass spectrometry imaging (MSI) have revealed how lipid metabolism patterns shape neuroplasticity and behavioral adaptation in fish. Lam et al. (2022) identified key modifications in golden barb brains, including enhanced oxidative phosphorylation, reduced DHA biosynthesis, and 5‐HT neuron demyelination, which may facilitate adaptation to cave environments. Similarly, the angiopoietin‐1 (Ang‐1) gene has emerged as a critical regulator of neuronal development and cognitive evolution in zebrafish (Chen et al. 2015).

Variations in habitats give rise to diverse phenotypes across various species of *Triplophysa*. Leveraging high‐throughput sequencing technology enables comprehensive exploration of the physiological nuances among distinct environments and species within the *Triplophysa* genus. Furthermore, it facilitates elucidation of the primary differentiating factors in physiological attributes between sympatrically distributed cave‐dwelling and surface‐dwelling variants of *Triplophysa*.

**FIGURE 1:**
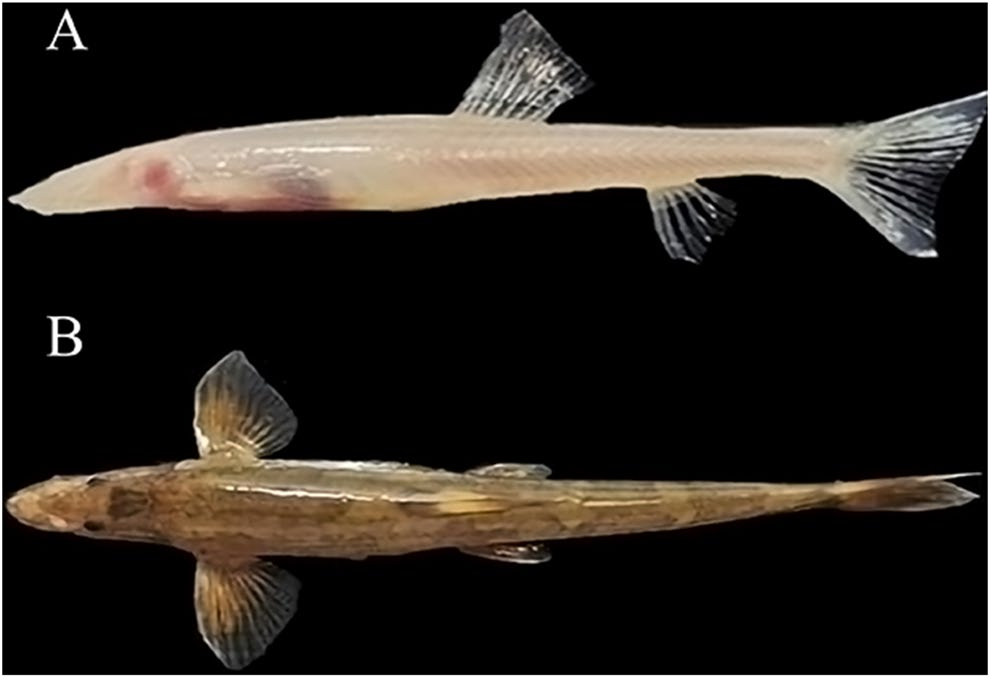
Morphology of two *Triplophysa* loaches. (A) *T. shilinensis* ; (B) *T. xiangshuingensis*.

In this investigation, our focus centered on the sympatric cave‐dwelling *T. shilinensis* and the surface‐dwelling *T. xiangshuingensis*. The distinct phenotypes of the two ecotypes are shown in Figure 1. Employing transcriptome sequencing technology, we conducted a comprehensive comparative analysis of tissue gene expression between these two distinct species of *Triplophysa*. The primary focus of this study involved the initial characterization of gene expression alterations within two *Triplophysa* tissues exhibiting varying degrees of adaptation to cave environments. The overarching objectives were as follows: (i) to acquire high‐fidelity brain transcriptome profiles of cave‐dwelling and surface‐dwelling species within the *Triplophysa* genus using Illumina sequencing; (ii) to discern differentially expressed genes (DEGs) through comparative analysis of brain transcriptome profiles between cave‐dwelling and surface‐dwelling species; and (iii) to pinpoint and validate pivotal candidate genes implicated in cave adaptation and developmental processes. This was achieved through qRT‐PCR experiments and comprehensive pathway and functional annotation analyses of DEGs, aimed at identifying and confirming key candidate genes crucial for cave adaptation and developmental phenomena across two distinct fish ecotypes.

Based on the selective pressures exerted by cave environments on fish physiological functions and the known phenotypic divergences in *Triplophysa*—such as eye degeneration and enhanced olfaction in cave morphs—this study proposes the following hypothesis: differentially expressed genes in the brains of the cave‐adapted *T. shilinensis* and the surface‐adapted *T. xiangshuingensis* will be significantly enriched in three functional categories—(1) energy metabolism and insulin signaling pathways, expected to be downregulated in the cave morph to reduce energetic costs; (2) visual development pathways, likely downregulated in response to perpetual darkness; and (3) olfactory signaling pathways, predicted to be upregulated to compensate for visual loss. Furthermore, the differential gene expression patterns between these morphs are hypothesized to converge with those observed in other cavefishes, reflecting a common molecular mechanism underlying cave adaptation.

## 2 Material and Methods

### 2.1 Sample Collection and Processing

The two species of *Triplophysa* under investigation, namely the cave‐dwelling *T. shilinensis* and the surface‐dwelling *T. xiangshuingensis*, were sourced from subterranean caves located in Gaoshishao Village, Shilin County, Kunming City, Yunnan Province, China on January 25, 2021. *T.shilinensis* inhabits subterranean river caves, while *T. xiangshuingensis* dwells in surface streams (Figure 1). Each species was represented by five biological replicates. Both specimens represent distinct phenotypes within *Triplophysa*, sharing the same geographical distribution yet exhibiting contrasting physical attributes. Detailed information regarding the *Triplophysa* samples is provided in Table 1.

**TABLE 1:** Collection information of the samples.

Sample name	Type	Number	Organization
*T. shilinensis*	Cavefish	SML‐1	Holistic organization
*T. shilinensis*	Cavefish	SML‐2	Holistic organization
*T. shilinensis*	Cavefish	SML‐3	Holistic organization
*T. shilinensis*	Cavefish	SML‐5	Holistic organization
*T. shilinensis*	Cavefish	SML‐6	Holistic organization
*T. xiangshuingensis*	Surface‐fish	XSQ‐1	Holistic organization
*T. xiangshuingensis*	Surface‐fish	XSQ‐2	Holistic organization
*T. xiangshuingensis*	Surface‐fish	XSQ‐3	Holistic organization
*T. xiangshuingensis*	Surface‐fish	XSQ‐4	Holistic organization
*T. xiangshuingensis*	Surface‐fish	XSQ‐5	Holistic organization

Abbreviations: SML, *T. shilinensis* ; XSQ, *T. xiangshuingensis*.

The live loach specimens were carefully placed into a fish fry container within a light‐free environment upon collection. To prevent any mortality during transportation and to ensure the preservation of their live state, we promptly utilized dissecting tools to conduct dissections immediately following collection, thereby providing an accurate representation of the specimens' condition. Throughout the collection and processing of loach samples, a systematic approach was employed. Initially, the live loach specimens were meticulously cleansed using 95% alcohol, followed by dissection utilizing sterilized dissecting scissors to expose the cranial region and extract the brain tissues. Subsequently, the surface of the brain was carefully wiped with alcohol and rinsed using 0.9% sterile saline. The excised brain tissues were then delicately transferred into sterilized cryopreservation tubes employing sterile forceps. These tubes, containing the brain tissues, were promptly immersed in liquid nitrogen tanks for immediate preservation. After the specimens arrived at the laboratory, they were further stored in a refrigerator at −80°C for subsequent analysis.

### 2.2 RNA Extraction, Library Construction and Illumina Sequencing

In the present investigation, the isolation of total RNA from the cerebral tissues of *Triplophysa* was accomplished through the utilization of the Trizol reagent (Ambion, USA).

RNA integrity was verified by electrophoresis on a 1% agarose gel. Concentration was quantified using the Qubit RNA Assay Kit with the Qubit 2.0 Fluorometer (Life Technologies, USA). Moreover, to conduct a thorough and rigorous evaluation of RNA quality, an assessment of RNA integrity, along with the determination of the RNA integrity number (RIN), was executed using the Agilent RNA 6000 Nano Kit (Agilent Technologies, USA) in conjunction with the Agilent Bioanalyzer 2100 system (Agilent Technologies, USA).

Using the high‐quality RNA obtained, a sequencing library was constructed. mRNA was isolated and purified from total RNA using oligo (dT) magnetic beads. The enriched mRNA was then randomly fragmented into short segments averaging approximately 200 base pairs (bp) in length. Upon enrichment, the mRNA fragments were subjected to stochastic fragmentation, yielding short fragments of around 200 bp. Subsequently, under the catalytic action of reverse transcriptase and in the presence of primers, these fragments underwent reverse transcription to generate double‐stranded complementary DNA (cDNA). The synthesized double‐stranded cDNA was then purified using DNA clean Beads. The ends of the purified cDNA were repaired with an End Repair Mix, followed by the addition of polyadenylate (poly (A)) tails to the 3′ end. This poly (A) tail addition was performed to enhance the efficiency of ligation to the sequencing adaptor. The ligated product was further purified and carefully selected as the template for polymerase chain reaction (PCR) amplification. The PCR product was fractionated and purified to obtain the final cDNA library. The purified library was then sequenced on an Illumina NovaSeq 6000 platform.

### 2.3 De Novo Assembly Analysis of Data

FastQC (Cock et al. 2010) and Trimmomatic (Bolger et al. 2014), quality control software tools, were deployed to eradicate adapters and substandard–quality reads from the Illumina platform's Fastq sequences, affirming the quality control of raw data. Subsequently, Fastq sequences were trimmed in accordance with base quality scores and filtered to produce high‐quality sequence data (Clean Data). De novo assembly of the Clean Data was conducted using the Trinity assembly software (Grabherr et al. 2011), culminating in the generation of the transcript Trinity fasta. Thereafter, the quality of the assembled transcripts was appraised using the Benchmarking Universal Single–Copy Orthologs (BUSCO) quality assessment software (Simão et al. 2015). The transcripts derived from splicing were further processed and clustered by means of the Corset hierarchical clustering software (Davidson and Oshlack 2014). Next, the longest transcript among the spliced transcripts was designated as the Unigene for in–depth subsequent analysis.

### 2.4 Bioinformatic Analysis

The RNA‐seq data were subjected to denovo splicing and bioinformatic analysis utilizing DESeq2, a software package that is based on the negative binomial distribution (Love et al. 2014). The criteria for screening differentially expressed genes (DEGs) were set as |log2 (fold change) | > 1 and adjusted *p*‐value < 0.05.

### 2.5 Validation of qRT‐PCR for Differential Genes

Owing to limitations in the quantity of fish samples collected, validation of all differentially expressed genes (DEGs) was not practicable. To evaluate the validation rate across the entire DEG set, six genes were selected using stratified random sampling for qRT‐PCR validation, representing both up‐ and down‐regulated genes across diverse functional categories.

The housekeeping gene β‐actin was employed as the internal reference gene; meanwhile, primers for the validated genes were designed utilizing Primer software (Zhai et al. 2008). Detailed primer designs are provided in Table 3. After the reaction was completed, the Ct values of the target genes were normalized utilizing the β‐actin internal reference gene as a standard. Subsequently, differential expression analysis of the target genes in each *Triplophysa* sample was performed using the relative quantitative 2^−∆∆Ct^ method (Livak and Schmittgen 2001).

## 3 Results

### 3.1 Sequencing and Assembly

Prior to library preparation and high‐throughput sequencing, the concentration, integrity (RNA Integrity Number [RIN]), and other key quality metrics of all RNA samples were systematically examined, and their overall quality was comprehensively evaluated. The quality control results of the RNA samples used in this study are summarized in Table 2. Comprehensive analysis of multiple indicators demonstrated that all ten RNA samples achieved a quality rating of Grade A, meeting the requirements for cDNA library construction in transcriptome sequencing.

**TABLE 2:** RNA sample quality assessment.

Species name	Sample ID	Concentration (ng/uL)	Volume (uL)	Total amount (ug)	RIN	Grade
	XSQ‐1	157	35	5.495	8.5	A
	XSQ‐2	86	35	3.01	8.6	A
*T. xiangshuingensis*	XSQ‐3	35	35	1.225	7.6	A
	XSQ‐4	128	35	4.48	8.2	A
	XSQ‐5	50	35	1.75	8	A
	SLM‐1	97	35	3.395	7.7	A
	SLM‐2	73	35	2.555	8.2	A
*T. shilinensis*	SLM‐3	90	35	3.15	7.1	A
	SLM‐5	31	35	1.085	7.2	A
	SLM‐6	57	35	1.995	8.1	A

**TABLE 3:** qPCR primers for differential genes.

Gene	Primer name	Sequence (5′—> 3′)	Length (bp)
*GLUT1*	HF‐GLUT1‐F	5′ CTGGGCATTGTTATTGGC 3′	164
	HF‐GLUT1_R	5′ ATGAGGAGGTAGCGTGGG 3′	
*CLOCK*	HF‐KAT13D‐F1	5′ ACCGACAGATAAGGTTTCC 3′	164
	HF‐KAT13D‐R1	5′ CTGGGTGATGCTGATTGT 3′	
*PRKCA/PKCa*	HF‐PRKCA‐F	5′ CTTTCATTTCTTGGCTCT 3′	148
	HF‐PRKCA_R	5′ CTCCACCATCGTACACTC 3′	
*RDH8*	HF‐RDH8‐F	5′ ACGCTGTTGGCTTTGGAT 3′	237
	HF‐RDH8‐R	5′ GATGATGTGCCCGCTTCG 3′	
*NPY2R*	HF‐NPY2R‐F	5′ GGAAAGGTCTCATCCGAACA 3′	146
	HF‐NPY2R‐R	5′ TGCCCACTAAGCCCACAG 3′	
*IRS1*	HF‐IRS1‐F	5′ GAAGCAAATCCCAAACTG 3′	212
	HF‐IRS1‐R	5′ AATACTGGCTGTCCGTGA 3′	
*β‐Actin*	β‐actinF	5′ GACCACCTTCAACTCCAT 3′	
	β‐actinR	5′ ACCACCAGACAATACAGT 3′	

**TABLE 4:** Brain transcriptome sequencing data of *Triplophysa*.

Sample	Raw reads	Clean reads	Clean bases	Error rate	Q20%	Q30%	GC content
SML_1	21594527	20691615	5.88G	0.02	98.11	94.36	41.53
SML_2	21744052	20405493	5.90G	0.02	98.33	94.9	41.94
SML_3	20785682	20159111	5.92G	0.03	97.88	93.86	40.91
SML_5	20967207	20519923	5.94G	0.03	98.03	94.08	41.11
SML_6	20423670	19673793	6.05G	0.02	98.47	95.14	41.68
XSQ_1	21695617	20835870	6.12G	0.02	98.11	94.31	42.09
XSQ_2	20327222	19588557	6.16G	0.02	98.15	94.42	42.92
XSQ_3	21819448	20746137	6.21G	0.02	98.32	94.87	42.5
XSQ_4	20518155	19783855	6.22G	0.02	98.44	95.12	43.44
XSQ_5	20546906	19718865	6.25G	0.02	98.39	94.98	42.95

**TABLE 5:** Statistical summary of the unigene set assembled by combining samples from both *T. shilinensis* and *T. xiangshuingensis*.

Length range	All transcript	All unigene
301–500	76,648	42,643
501–1000	64,388	28,083
1001–2000	59,418	13,730
> 2000	87,643	17,269
Total unmber	288,097	101,725

**TABLE 6:** Key representative differentially expressed genes involved in major adaptive pathways of *T. shilinensis* and *T. xiangshuingensis*.

Gene name	Pathway	Regulate	log2FC
*ADCY3*	Olfactory transduction	Up	1.8729
*CRY*	Circadian rhythm	Down	−2.4752
*CLOCK*	Circadian rhythm	Down	−1.9905
*GLUT1*	Insulin secretion	Down	−2.7095
*ADCY9*	Melanogenesis	Down	−1.3674
*RDH8*	Retinol metabolism	Down	−1.6726
*IRS1*	Insulin signaling pathway	Down	−1.8962
*NPY2R*	Neuroactive ligand‐receptor interaction	Down	−2.2805
*LRAT*	Retinol metabolism	Down	−2.6625
*GHR*	Neuroactive ligand‐receptor interaction	Down	−2.3421
*PACAPRI*	Neuroactive ligand‐receptor interaction	Down	−4.2574
*GHRHR*	Neuroactive ligand‐receptor interaction	Down	−1.2946
*ACSL*	Fatty acid degradation	Down	−2.4252
*CNGB1*	Phototransduction	Down	−1.9245
*PIGR*	Intestinal immune network for IgA production	Down	−1.9596

In this study, sequencing was performed using the Illumina NovaSeq 6000 platform, which generated Raw Data through high‐throughput sequencing. Consequently, Clean Data was derived by filtering out reads with subpar sequencing data quality, followed by quality assessment and base distribution statistics analysis. Following sequencing quality control, a total of 60.74 Gb of clean data was acquired; the results of the comprehensive assessment of the quality of the brain tissue samples obtained from the plateau loach are presented in Table 4.

The clean reads underwent splicing utilizing the Trinity splicing software, with the longest transcript selected from the assembled transcripts and designated as Unigene. The transcriptome analysis of *Triplophysa* brain tissue unveiled 288,097 transcripts and 101,725 Unigenes in the library. Comprehensive information regarding the sequence and length distribution of transcripts and Unigenes is presented in Table 5.

### 3.2 Functional Annotation for Unigenes

**FIGURE 2:**
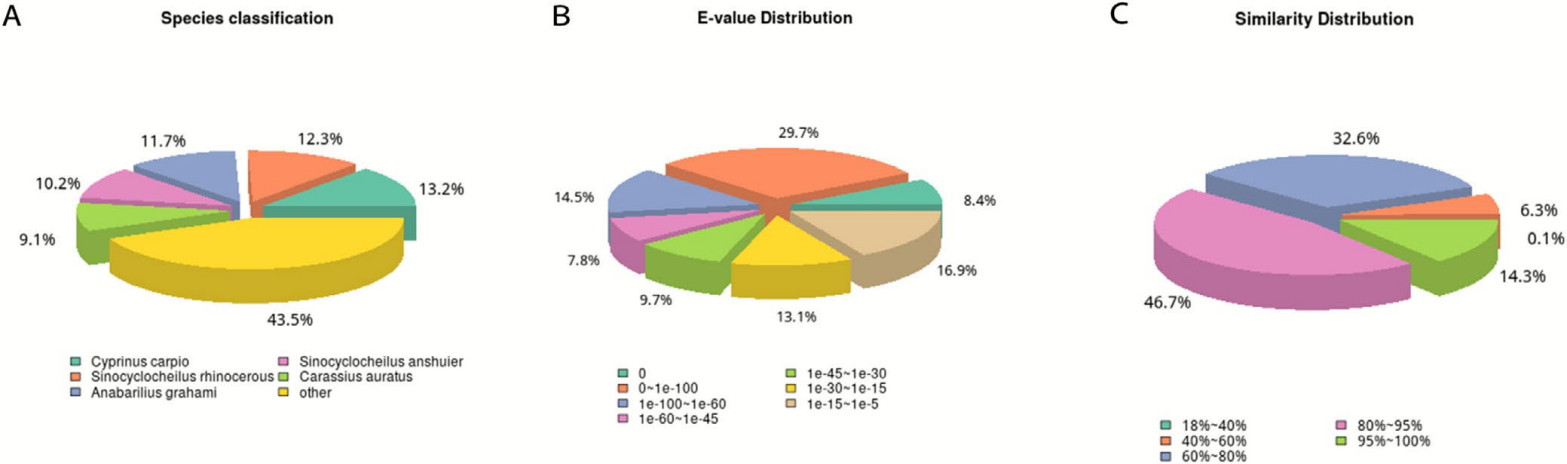
(A) The distributions of species for *T.shilinensis* and *T*. *xiangshuingensis* based on BLAST against Nr databases. (B) The distributions of E‐value for *T. shilinensis* and *T. xiangshuingensis* based on BLAST against Nr databases. (C) The distributions of similarity for *T. shilinensis* and *T. xiangshuingensis* obtained from BLAST searches against Nr databases.

In this study, sequence annotation was performed by aligning unigenes against the NR (Non‐Redundant) protein database from NCBI using BLASTX with an *E*‐value cutoff of 1e‐5, which led to the annotation of 35,802 unigenes. We conducted an analysis of homologous species within the NR database and generated graphs depicting the classification, value distribution, and probability distribution of species annotated to the NR database by unigene. These plots are provided in Figure 2A–C. As depicted in Figure 2A, the comparison between the two species of *Triplophysa* encompassed several species, notably including carp, *Sinocyclocheilus rhinocerous* , *Anabarilius grahami* , *Sinocyclocheilus anshuiensis*, and *Carassius auratus* . These annotated species serve as crucial references for conducting further analysis of gene expression within the brain tissue of *Triplophysa* species. As illustrated in Figure 2B, it is evident that an E‐value of 0 accounted for only 8.4% of the total, indicating that the comparison of unigenes from the transcriptomes of the two *Triplophysa* species to the NR database was highly reliable. As observed in Figure 2C, unigenes exhibiting similarity scores ranging from 60% to 100% comprised 93.6% of the total unigenes, suggesting that the results of the comparison between the blind *T. shilinensis* and the *T. xiangshuingensis* turnip to the NR database were highly reliable. A total of 10,855 genes were annotated in the KOG database.

**FIGURE 3:**
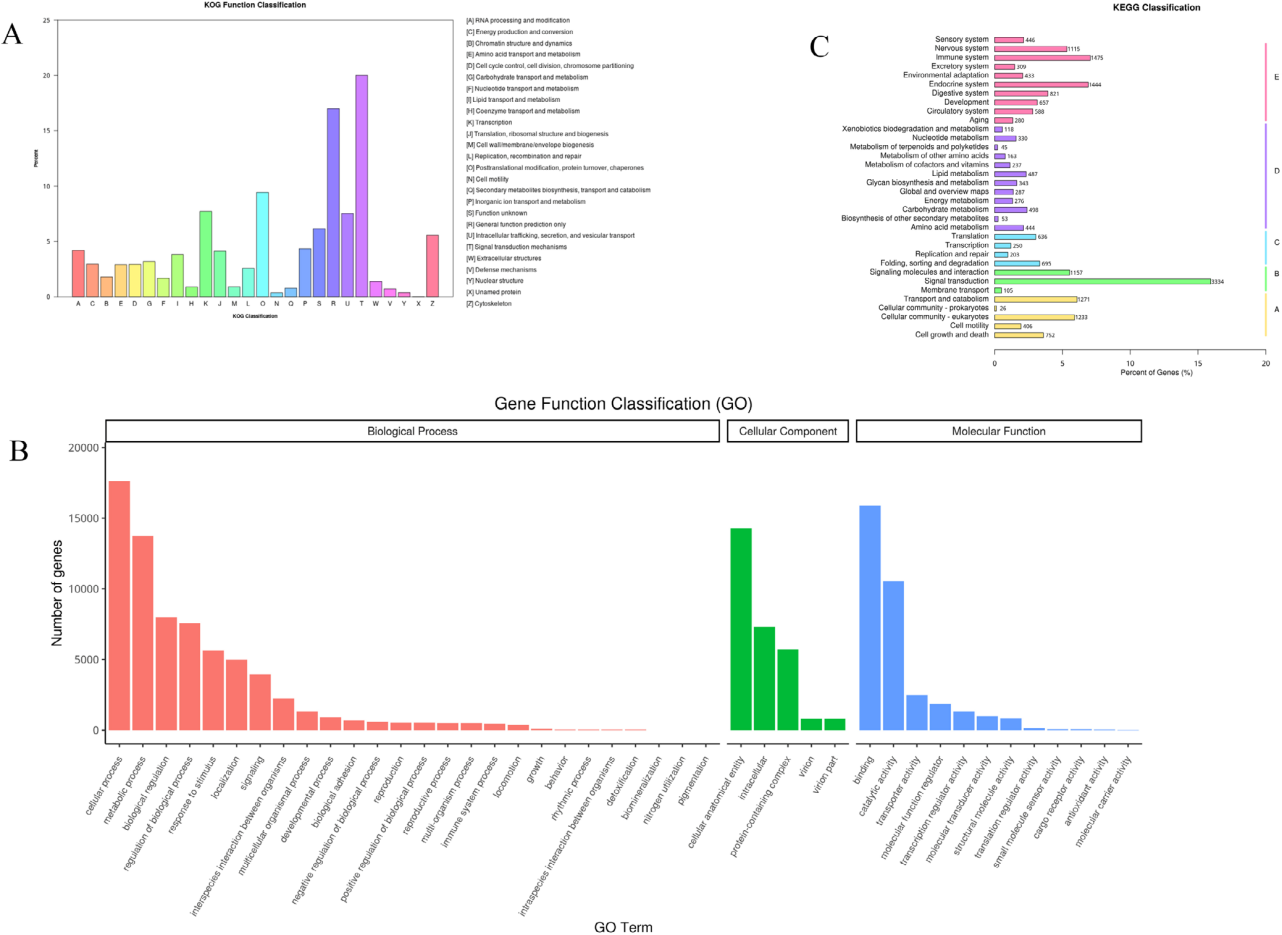
(A) The distributions of species for *T. shilinensis* and *T. xiangshuingensis* based on BLAST against Nr databases. (B) GO functional annotation statistics: The horizontal axis in the Fig is the GO level 2 functional classification unit, and the vertical axis is the number of Unigenes annotated to that functional classification unit. (C) The horizontal axis of the graph indicates the number of genes annotated within the relevant pathway and the ratio of the number of these genes to the total number of genes annotated for that pathway. The vertical axis represents the names of the metabolic pathways.

Through the KOG functional classification map (Figure 3A), it was observed that the Unigenes within the brain tissue transcriptomes of *T. shilinensis* and *T. xiangshuingensis* were annotated across various functional categories in the KOG database. Specifically, a substantial number of Unigenes were annotated to categories such as Signal transduction mechanisms, General function prediction only, Posttranslational modification, protein turnover, chaperone‐related processes, Intracellular trafficking, vesicular transport, and secretion pathways. In contrast, a significantly smaller number of Unigenes were annotated to functional categories such as nuclear structure, Cell motility, Defense mechanisms, Secondary metabolite biosynthesis, transport and catabolism, and Coenzyme transport and metabolism.

To gain a more comprehensive understanding of the functions assigned to the identified Unigenes, a Gene Ontology (GO) based analysis was carried out. The brain transcriptomes of the two *Triplophysa* species investigated in this study were subjected to Gene Ontology (GO) analysis, which encompasses three primary categories: Biological Processes, Cellular Components, and Molecular Functions. A total of 28,854 Unigenes were successfully annotated in the GO database, furnishing functional information across 1351 levels of annotation. When mapping the Unigenes annotated to level 2 of the GO hierarchy (Figure 3B), it was found that there were 27, 5, and 12 level 2 GO terms annotated within Biological Processes, Cellular Components, and Molecular Functions, respectively. These functional annotations are pivotal for gaining deeper insights into gene expression patterns within *Triplophysa* brain tissues.

The KEGG pathway database was employed to identify biological pathways associated with the function of Unigenes. In total, 8356 Unigenes were annotated following KEGG pathway analysis of the transcriptomes derived from the brain tissues of the two *Triplophysa* species examined in this study. As illustrated in the KEGG metabolic pathway classification diagram (Figure 3C), the KEGG metabolic pathways annotated by the Unigenes in this research were categorized into several categories, namely Cellular Processes, Environmental Information Processing, Genetic Information Processing, Metabolism, and Organismal Systems. These categories are denoted by branches labeled A, B, C, D, and E, respectively, in Figure 3C. Specifically, the Organismal Systems branch (branch E) encapsulated 568 genes, the Genetic Information Processing branch (branch B) contained 1784 genes, and the Environmental Information Processing branch (branch D) contained 3360 genes.

### 3.3 Differential Expressed Gene Analysis

**FIGURE 4:**
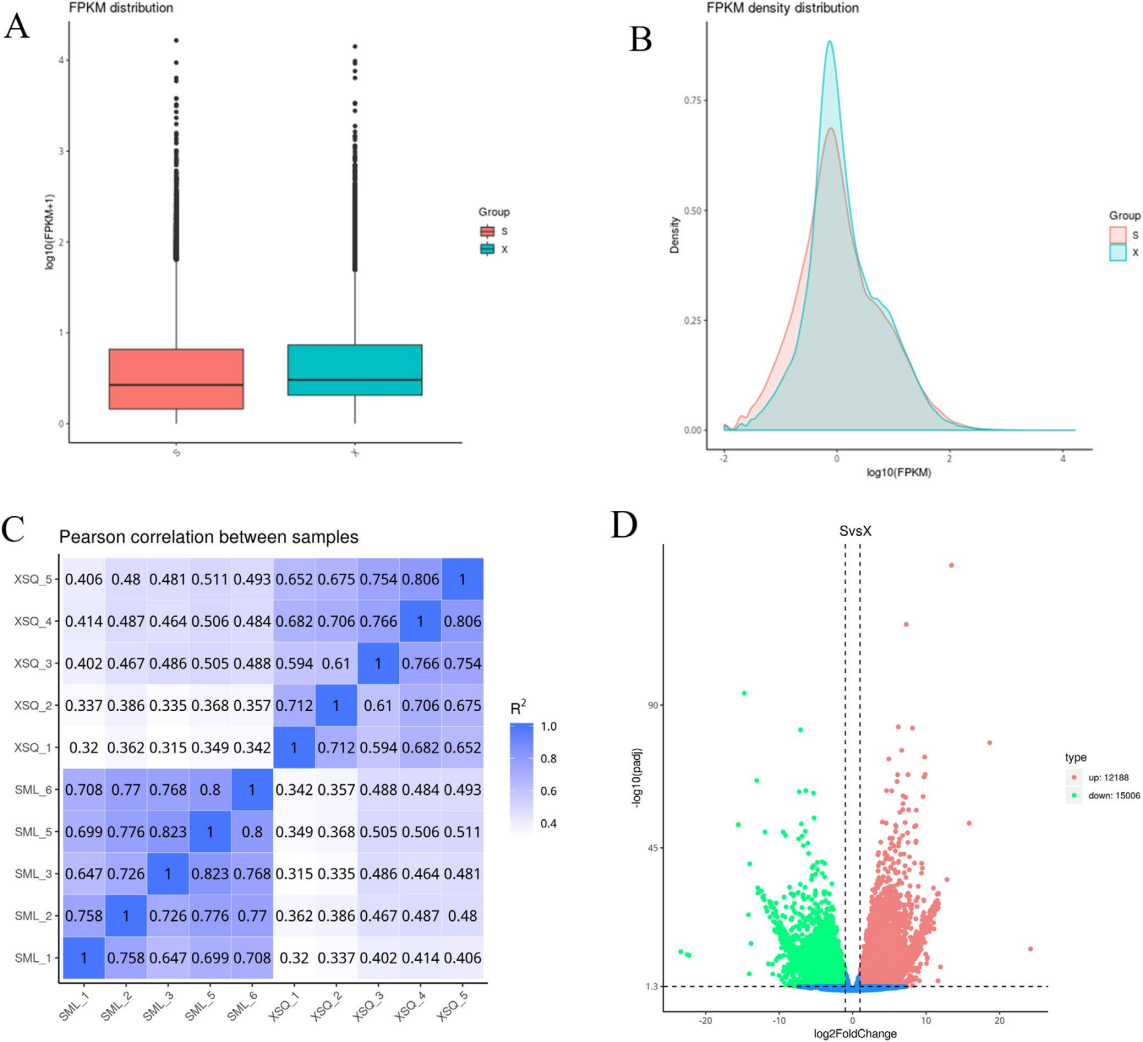
(A) The horizontal axis represents the sample names, S: *T.shilinensis*, X: *T. xiangshuingensis*, and the vertical coordinates are log10 (FPKM+1) box plots for each region for the five statistics for the maximum, upper quartile, median, lower quartile, and minimum values from top to bottom. (B) Density distribution of the FPKM between *T. shilinensis* and *T. xiangshuingensis*, S: *T. shilinensis* , X: *T. xiangshuingensis* the horizontal axis is log10 (FPKM), and the vertical coordinate is the density of genes. (C) Analysis of correlation between samples. (D) Differentially expressed gene volcano plot: The horizontal coordinate is the fold change in gene expression and the vertical coordinate is the negative logarithm of the *p*‐value of the significance test −log10 *p*‐adj.

The gene expression levels in the brain transcriptome of *Triplophysa* were quantified by the FPKM method (Figure 4A,B). An analysis of these Figs reveals significant deviations in both the maximum and median gene expression levels among brain tissues of *T. shilinensis* and *T. xiangshuingensis*. In the density distribution map of FPKM, remarkable differences in gene expression were detected between the brain tissues of the two *Triplophysa* species. Specifically, the density of certain genes in *T. xiangshuingensis* was distributed above 0.75, whereas the density of genes in *T. shilinensis* did not surpass this threshold. This finding suggests a significant disparity in gene expression profiles among the brain tissues of distinct phenotypes of *Triplophysa* species. The correlation of gene expression levels across samples can act as an important reference for assessing sample selection and validating the reliability of the experimental results. A greater similarity in expression patterns between samples is indicative of a correlation coefficient that approaches closer to 1. As shown in the correlation analysis graph between the two *Triplophysa* species in this study (Figure 4C), differences in gene expression patterns were observable between brain tissues of these two *Triplophysa* species.

The experimental group comprised the cave‐type *T. shilinensis* , which was composed of the control group made up of the surface‐type *T. xiangshuingensis*. The number of differentially expressed genes was ascertained, and their expression levels were visualized via a statistical graph and volcano plot (Figure 4D), in total, 27,194 differentially expressed genes were identified between the brain tissue samples of *T. shilinensis* and *T. xiangshuingensis*. Among these genes, 15,006 genes were down‐regulated, whereas 12,188 were up‐regulated.

**FIGURE 5:**
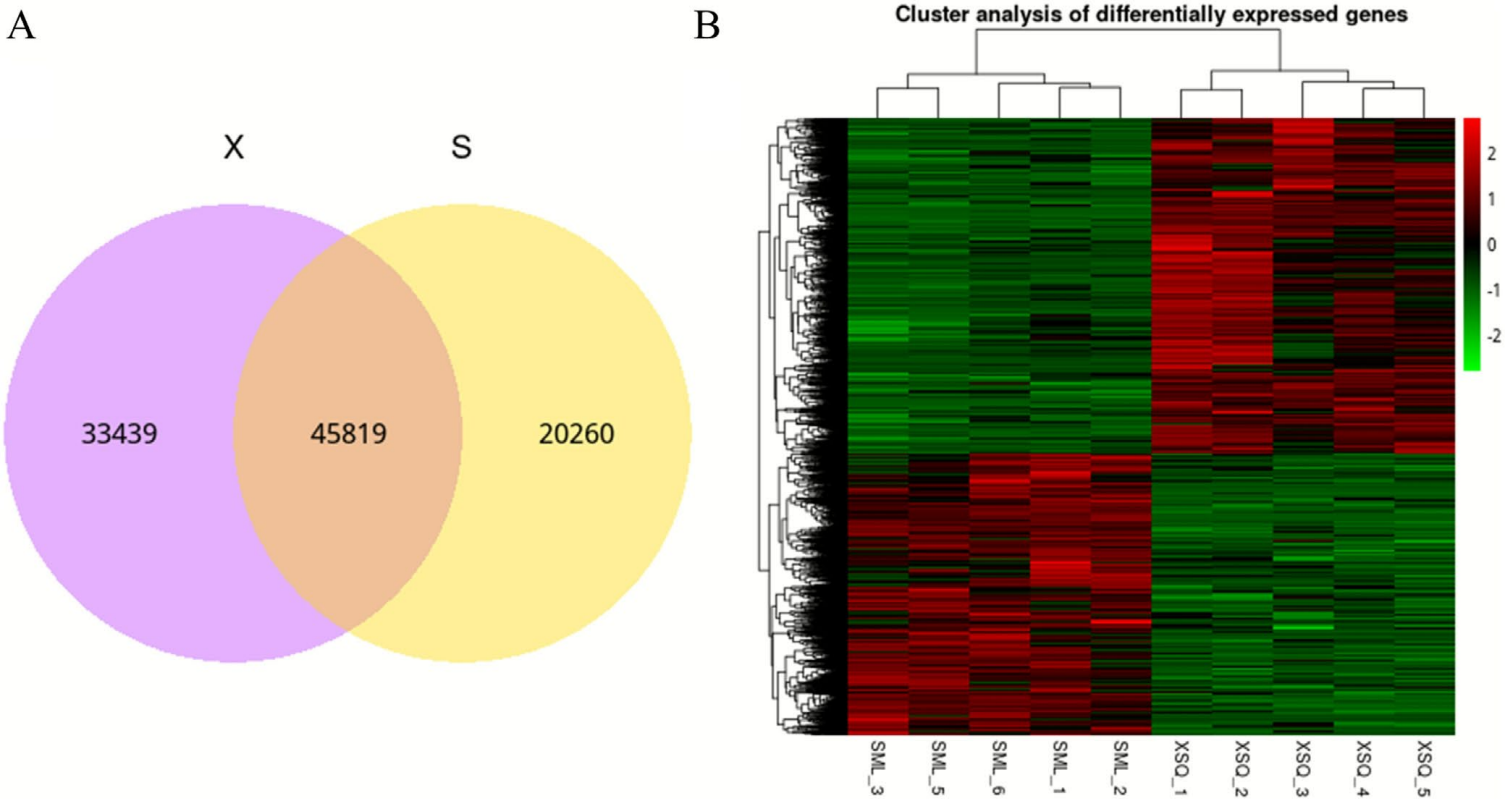
(A) Venn diagram showing the number of genes expressed (FPKM ≥ 1) exclusively in the surface‐dwelling *T. xiangshuingensis* (control, 33,439), exclusively in the cave‐dwelling *T. shilinensis* (experimental, 20,260), and shared between both ecotypes (45,819). (B) Clustering heatmap of differentially expressed genes (red: highly expressed genes; green: lowly expressed genes).

A Venn diagram of differentially expressed genes was generated with respect to the genes recognized as differentially expressed in the two *Triplophysa* species (Figure 5A). As is apparent from the Fig, it is evident that significant differences exist in the expression patterns within the brain tissues of *T. xiangshuingensis* and *T. shilinensis* . Specifically, a total of 33,439 genes were exclusively expressed in the surface‐dwelling control group (*T. xiangshuingensis*), while 20,260 genes were specific to the cave‐adapted group ( *T. shilinensis* ). Additionally, a total of 45,819 genes were shared between *T. xiangshuingensis* and *T. shilinensis*. The total number of expressed genes (99,518) is lower than the assembled Unigenes (101,725) because not all transcripts were expressed above the detection threshold (FPKM ≥ 1) in the analyzed tissues.

The screened differential gene sets were subjected to clustering analysis, yielding a heat map that depicts the clustering of differentially expressed genes (Figure 5B). From the heat map, it is readily apparent that the experimental group of *T. shilinensis* clustered cohesively as one distinct category, while the control group of *T. xaingshuijingensis* clustered separately as another category, without any inter‐crossover phenomenon. This observation strongly suggests the high accuracy and reliability of the transcriptome sequencing data obtained in this study. Furthermore, the consistency among the five biological samples of the same *Triplophysa* species was relatively high, indicating potential differences in expression patterns between the cave‐type *T. shilinensis* and the surface‐type *T. xaingshuijingensis*.

### 3.4 GO Enrichment Analysis of Differential Genes

**FIGURE 6:**
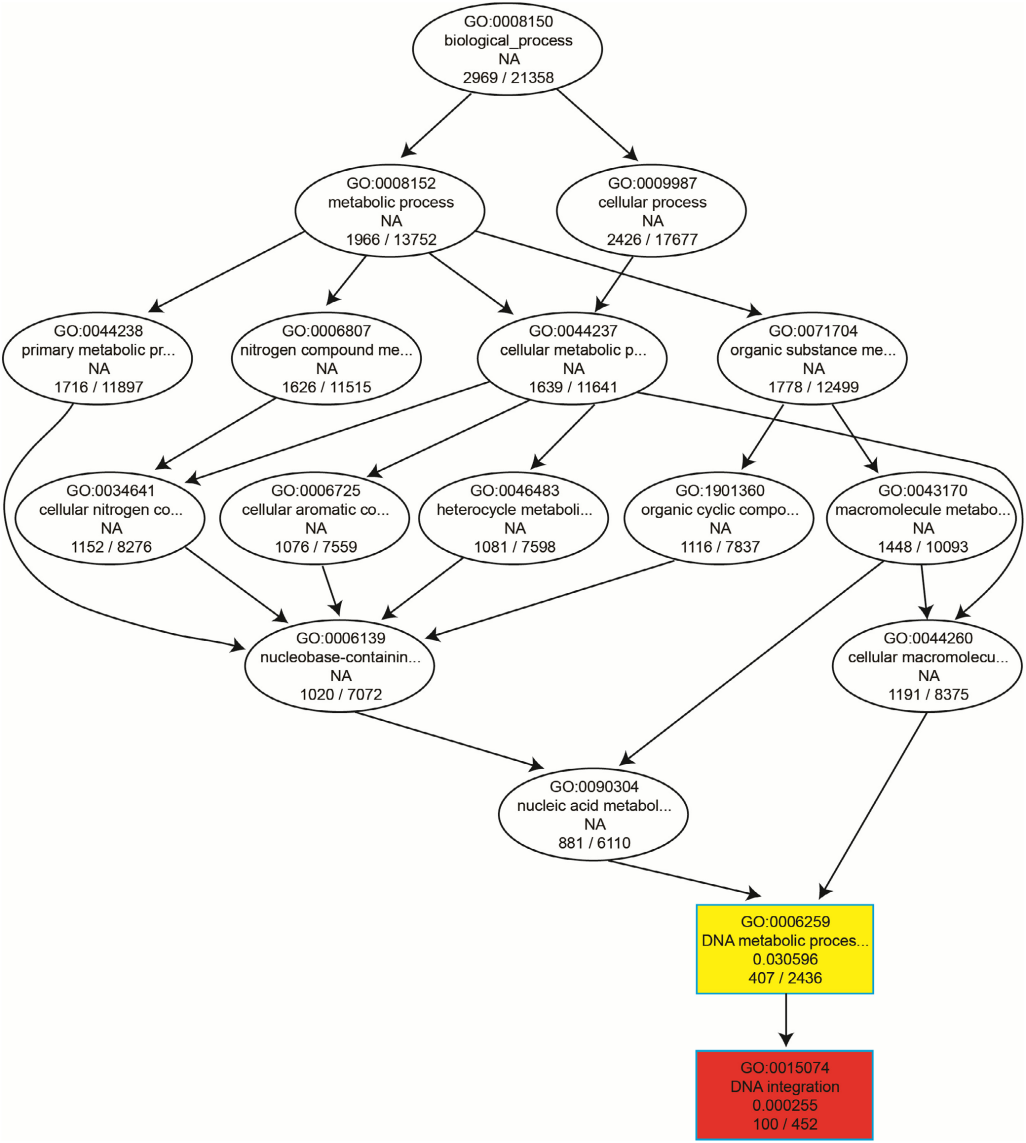
GO enrichment directed acyclic graph.

In the GO enrichment analysis, the up‐regulated differentially expressed genes in the brain tissues of *Triplophysa* were exclusively enriched in the biological processes of “DNA integration” (GO: 0015074) and “DNA metabolic process” (GO: 0006259), with 100 and 407 GO entries, respectively, as detailed in Figure 6. This finding suggests significant differences in the growth and development processes between *T. shilinensis* and *T. xiangshuingensis*.

## 4 KEGG Enrichment Analysis of Differential Genes

**FIGURE 7:**
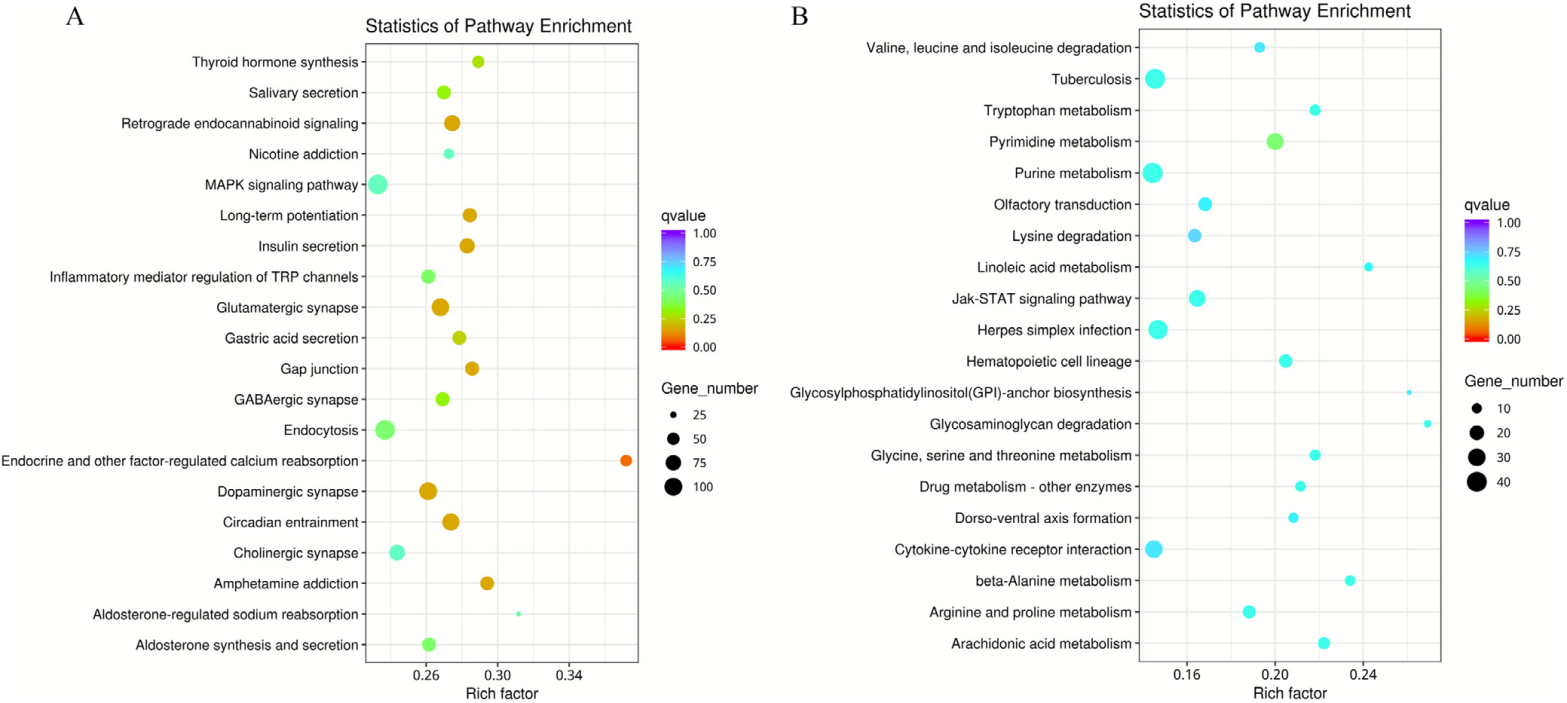
(A) Bubble chart of KEGG enrichment analysis for downregulated differentially expressed genes; (B) Bubble chart for upregulated differentially expressed genes.

The down‐regulated differentially expressed genes were analyzed using KEGG pathway enrichment analysis, and a bubble plot illustrating the enrichment of these genes was generated (Figure 7A). As shown in the Fig, it is clear that the enrichment of these down‐regulated differentially expressed genes is significant, with the enrichment degree concentrated within the range of 0.26–0.30. The pathways enriched by the differentially expressed genes include Glutamatergic synapse, Insulin secretion, Circadian entrainment, Gap junction, Dopaminergic synapse, MAPK signaling pathway, Thyroid hormone synthesis, Retrograde endocannabinoid signaling (Love et al. 2014), and Salivary secretion, among others. Notably, the pathway of the endocrine and other factors regulating calcium reabsorption exhibited the most pronounced and highest level of enrichment.

As delineated in the KEGG bubble plot of upregulated differentially expressed genes in this study (Figure 7B), the q‐value values for most pathways were concentrated within the interval of 0.5–0.75. Notably, Pyrimidine metabolism exhibited significant enrichment, with Glycosaminoglycan degradation emerging as the most highly enriched pathway. Additionally, significant enrichment was observed in pathways such as Purine metabolism, Herpes simplex virus infection, and cytokine–cytokine receptor interaction. Moreover, a substantial quantity of genes was found to be enriched in pathways including Olfactory transduction and Dorso–ventral axis formation. These findings underscore the potential involvement of these pathways in the upregulated gene expression patterns observed in the present investigation.

Hence, the predominant differentially expressed genes identified in KEGG‐enriched pathways within the brain tissues of the two *Triplophysa* species encompass genes associated with circadian rhythms, insulin secretion, retinol metabolism, neuroactive ligand‐receptor interactions, insulin signaling pathway, fatty acid degradation, melanin synthesis, olfactory conduction, and other metabolic pathways, as outlined in Table 6.

### 4.1 Selection of Target Differential Genes and Expression Trend Validation

**FIGURE 8:**
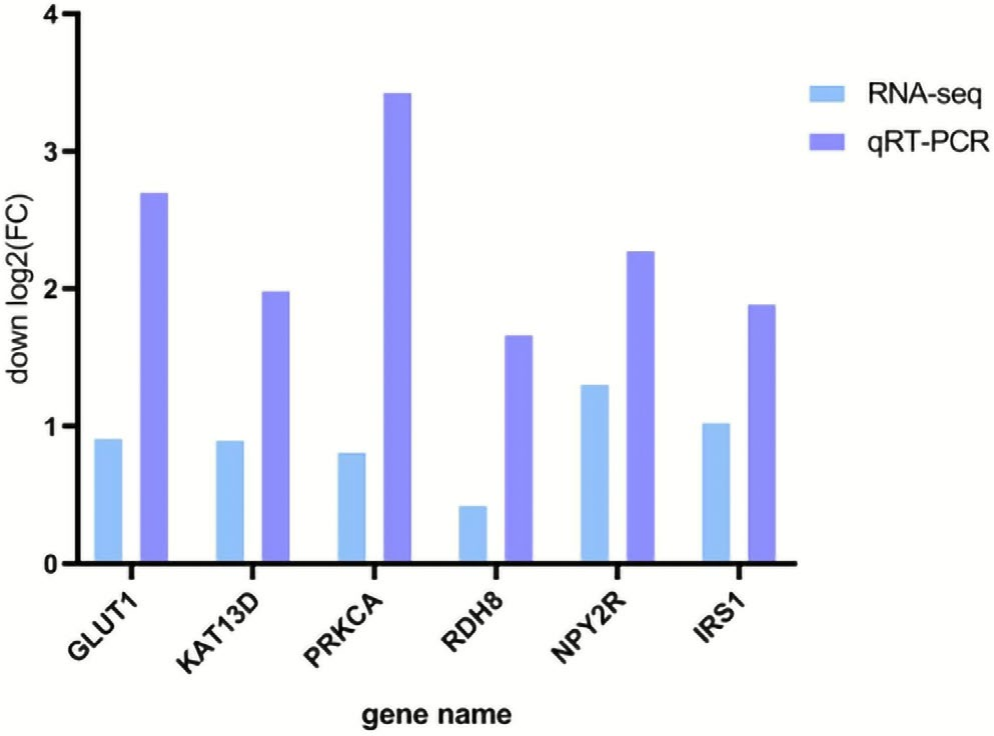
RT‐qPCR to verify RNA‐seq result. The abscissa represents the gene name, and the ordinate represents the fold change of downregulated differential genes (log_2_Fold Change).

Following the acquisition of transcriptomic data from the brain tissues of *T. shilinensis* and *T. xiangshuingensis*, we performed qRT‐PCR validation to assess the reliability of the RNA‐seq results. Six differentially expressed genes (DEGs) related to cave adaptation in *Triplophysa* (*GLUT1*, *PRKCA*, *RDH8*, *IRS1*, *NPY2R*, and *KAT13D*) were selected for expression‐level verification. The qRT‐PCR results indicated that, although the fold changes in expression differed somewhat from those in the transcriptomic data, the expression trends were consistently downregulated, demonstrating that the transcriptomic data obtained in this study is highly reliable. The validation results are shown in Figure 8.

## 5 Discussion

The unique subterranean ecosystems of caves have led to the evolution of the troglobionts in response to the chronic environmental constraints posed by the environment. Findings have established that goal–directed complex behaviors, including food foraging and predator evasion, along with emotionally–driven behaviors such as aggression, anxiety, and fear, are regulated by a neuromodulatory system centered in the brain (Guo 2004). Previous research on certain cavernicolous fish indicates that specific brain genes may significantly contribute to the evolution of behaviors tailored to cave habitats. For instance, mutations in the coding sequence of the MAO (monoamine oxidase) gene observed in the brain of *Astyanax mexicanus* could potentially elevate serotonin levels concomitantly with a reduction in monoamine oxidase activity. This genetic alteration is believed to have resulted in a complete loss of aggressive behavior and the emergence of novel foraging behaviors, which researchers propose aid in energy acquisition in *A. mexicanus* (Elipot et al. 2014; Elipot et al. 2013). In this study, to minimize the impact of natural environmental variables, we opted for two sympatric *Triplophysa* species, namely, distinct types of fish (burrowing and surface), inhabiting the same aquatic environment but at varying depths. Through comparative transcriptome analysis of brain transcriptome data obtained from the two *Triplophysa* species, we identified numerous candidates for differentially expressed genes (DEGs) from a total of 27,194 DEGs. These identified genes hold promise for elucidating the adaptations observed in cave‐dwelling *Triplophysa* fishes. A study by Zhao et al. demonstrated that, notwithstanding their different genera, *Sinocyclocheilus* and *Triplophysa* exhibit convergent evolution in adapting to cave environments. This suggests that cave‐living fishes share similar regulatory patterns of gene expression in their brain tissues as they adapt to the extreme conditions of caves (Li et al. 2020).

### 5.1 Down—Regulation of Genes Associated With Insulin Secretion and Energy Metabolism in *T. Shininess*

In vertebrates, the brain, 0.1%–1% of body mass, has high metabolic demand and regulates overall energy expenditure via the peripheral nervous system or hormones (Ginneken et al. 1996). Insulin, a crucial endocrine hormone in animals, exerts influence on brain activity across the lifespan. It regulates animal growth, development, and metabolism in response to material and energy requirements, sustaining life's stability. Fish brains are replete with insulin, which actively modulates brain energy metabolism (Soengas and Aldegunde 2002). Comparative studies reveal that cave‐adapted populations of *A. mexicanus* exhibit enhanced skeletal muscle glycogen metabolism relative to epibenthic fishes. Mechanistically, mutations in the insulin receptor led to insulin resistance in these cave‐dwelling specimens. The authors propose that maintaining elevated blood glucose levels in caves for extended durations could serve as a strategy to contend with intermittent food shortages (Olsen et al. 2023).

Xu et al. demonstrated that protein kinase C α (PRKCA/PKCα) is a key modulator of insulin secretion. Hyperglycemic conditions in pancreatic β‐cells stimulate PKCα activity, increasing *TRPC1* phosphorylation and facilitating insulin secretion. Conversely, pharmacological or genetic suppression of PKCα activity significantly attenuates glucose‐stimulated insulin secretion (GSIS), highlighting the significant relationship between PKCα activity and insulin secretion (Xu et al. 2019). In this study, PKCα gene expression was downregulated in the brain tissue of *T. shilinensis* compared to *T. xiangshuingensis*. This transcriptional attenuation of PKCα gene expression may contribute to the reduced insulin secretion observed in *T. shilinensis* . However, further functional experiments are required to validate this hypothesis. Insulin receptor substrate 1 (*IRS1*) serves as a key substrate for both insulin receptor and insulin‐like growth factor‐1 (*IGF‐1*) receptor tyrosine kinase. Its role encompasses the regulation of numerous insulin responses, notably in carbohydrate and lipid metabolism. Kamei et al. discovered that *IRS1* acts as a catalytic driver in mediating restricted insulin/insulin‐like growth factor signaling (*IIS*) in zebrafish during the early embryonic period. Their study proved that the loss of IRS1 expression significantly impedes the growth of zebrafish embryos (Kamei et al. 2018). Hayashi et al. verified that mice lacking IRS1 in the brain exhibited decreased expression of *GHRH* (hypothalamic growth hormone‐releasing hormone), leading to impaired neurite elongation. These mice displayed lower body weights, reduced body and bone lengths, diminished bone mineral density, and inhibited growth. This suggests that *IRS1* is pivotal in regulating synaptic growth, somatic growth, and glucose homeostasis in *GHRH* neurons via the hypothalamus (Hayashi et al. 2021). In the cave‐dwelling *T. shilinensis* , *IRS1*, a gene crucial for insulin secretion, was significantly downregulated. This downregulation potentially disrupts glucose conversion and utilization, energy metabolism, and growth processes, possibly contributing to the smaller size of *T. shilinensis* compared to the surface‐dwelling Rattlesnake loach. However, the precise influence of IRS1 downregulation on the growth and development of troglomorphic *T. shilinensis* warrants further comprehensive investigation.

Glucose transporter protein 1 (*GLUT1*/*SLC2A1*) serves as a linchpin energy transporter in the brain, primarily facilitating glucose transport across the blood–brain barrier and promoting glucose uptake within brain tissues (Geng et al. 2021). Deficiency of *GLUT1* during infancy in humans has been linked to developmental delays, acquired microcephaly, spasticity, ataxia, and hypoglycemia, highlighting its critical role in normal brain development and function (Verrotti et al. 2012). G*LUT1* exhibits ubiquitous expression across tissues in Atlantic cod, with particularly high expression levels observed in brain transcripts (Hall et al. 2004). In South American white shrimp, Jiao observed that continuous dark conditions led to suppressed *GLUT1* expression, consequently causing a decrease in blood glucose levels (Jiao et al. 2021). Jensen discovered that the elimination of *GLUT1* in early zebrafish embryos led to a suppression of glucose uptake inhibition, accompanied by increased neuronal apoptosis in the embryos (Jensen et al. 2006). *GLUT1* expression was downregulated in the brain tissue of *T. shilinensis* compared to *T. xiangshuingensis*. This downregulation may hinder glucose metabolism and impair energy production in *T. shilinensis* , suggesting a role for *GLUT1* in the energy metabolism of plateau loach that requires further verification. Lopes et al. identified the expression patterns of the *ACSL* family genes (*ACSL1*, *ACSL2*, *ACSL3*, *ACSL4*, *ACSL5*, *and ACSL6*) across various tissues in Scleractinia fish and zebrafish. Notably, *ACSL4* was found in cavefish, while *ACSL6* was predominantly observed in the brain tissue of zebrafish (Lopes‐Marques et al. 2013). *ACSL* (long‐chain acyl coenzyme A synthetase) serves as a pivotal enzyme in the initial stages of lipid metabolism. It functions by activating fatty acids (FAs), thereby directing acyl coenzyme A towards lipid anabolism or catabolism via β‐oxidation, a crucial process within energy metabolism (Zhao et al. 2019). In this investigation, *ACSL* was pinpointed within the fatty acid biosynthesis and fatty acid degradation pathways in the brain tissue transcriptome of the plateau loach. A notable decrease in *ACSL* expression was observed in the cave‐dwelling *T. shilinensis* , with the surface‐dwelling *T. xiangshuingensis* serving as the control. This suggests that the down‐regulation of *ACSL* expression might impact the energy metabolism of *T. shilinensis* within the dark cave environment.

### 5.2 Down‐ Regulation of Circadian Rhythm and Behavior Genes in *T. shilinensis*

Biological clocks, functioning as endogenous time–keeping mechanisms within organisms, are precisely synchronized with the environmental circadian cycle. These clocks modulate a diverse array of physiological and behavioral processes through the feedback loops of clock‐related factors. Such modulation has far–reaching impacts on crucial biological aspects, including metabolism, cell growth, and physiological functions (Takahashi 2017). In fish, multiple clock genes, including *PER* (Period), *CRY* (Cryptochrome), *CLOCK* (Circadian Locomotor Output Cycles Kaput), *BMAL* (Brain and Muscle ARNT–like protein), *ROR* (Retinoic acid–related orphan receptor), and *REVERB* (Rev–ERB), play pivotal roles in maintaining and regulating the circadian system (Zhdanova 2005). The *CRY* gene, along with its family members *CRY1* and *CRY2*, serves as transcriptional repressors essential for circadian photoreception and peripheral biological clock oscillations in clock cells (Lucas and Foster 1999). In Drosophila, deficiency in *CRY* leads to unresponsive circadian rhythms (Collins et al. 2006). *FBXL3* (F‐box/leucine‐rich‐repeat protein 3) regulates the degradation of *CRY* proteins, influencing the pace of molecular oscillations and consequent behavioral rhythms. Studies on zebrafish have shown that loss of *FBXL3A* function leads to diminished promoter activity, disruption of circadian mRNA expression rhythms, and disturbances in locomotor activity and sleep–wake cycles (Confino et al. 2022). He et al. observed that alterations in the expression levels of clock genes such as *PER*, *CRY*, *CLOCK*, *BMAL2*, *PDP (PAR–domain* protein), *DEC*(Differentiated embryo chondrocyte), and *FBXL3* could induce modifications in circadian rhythms among grass carp following a dietary shift (He et al. 2015). Moran discovered that the cave‐dwelling, eyeless *A. mexicanus* , deprived of light and temperature cues in its dark habitat, ceases to sustain circadian rhythms as a strategy for energy preservation (Moran et al. 2014). The findings of this study revealed a significant decrease in mRNA expression levels of *CRY*, *CLOCK*, and *FBXL3* clock genes in the brain tissue of cave‐dwelling *T. shilinensis* compared to surface‐dwelling Rattlesnake loach. It is hypothesized that the downregulation of these circadian rhythm‐related genes may dampen the circadian rhythm of *T. shilinensis* , which inhabits dark cave environments devoid of external circadian cues. This downregulation of circadian rhythm regulatory genes could potentially enhance its foraging efficiency in the absence of light cues. However, further comprehensive studies are required to validate this speculation.

Ahi et al. examined the expression of appetite‐regulating genes in the brains of herbivorous and carnivorous trophic niches of cichlid seabreams. The researchers observed that the anorexigenic gene *NPY2R* was upregulated in the brains of carnivorous cichlid seabreams but reduced expression in herbivorous counterparts. This disparity suggests a compensatory mechanism in herbivorous species, potentially to counterbalance their less nutritious diets compared to carnivorous counterparts (Ahi et al. 2020). In this investigation, *NPY2R* in the brain tissue of cave‐dwelling *T. shilinensis* was decreased compared to surface‐dwelling rattlesnake plateau loach. The reduction in *NPY2R* expression in *T. shilinensis* reflects adaptation to its dark and food‐scarce environment. This down‐regulation signifies a strategy to maintain a higher appetite level and increased feeding activity to cope with limited food resources. Further experimental validation is required to confirm the role of *NPY2R* in regulating the feeding behavior of blind *T. shilinensis* .

### 5.3 Down‐ Regulation Genes Involved in Color Development in *T. shilinensis*

Skin pigmentation is one of the most diversified phenotypic traits in fish, resulting from complex biological processes regulated by genetic factors. Fish body color is influenced by environmental, nutritional, physiological, and genetic conditions. The melanogenesis pathway plays a pivotal role in fish body color development (Chen et al. 2020). Jiang et al. demonstrated that *GNQA* in the melanin synthesis pathway is associated with the body color of both Xingguo red carp (*Cyprinus carpio var. xingguonensis*) and yellow carp (*Cyprinus carpio haematopterus*). The study conducted by Jiang et al. (2014), revealed that the expression of *GNQA*, a component within the melanin synthesis pathway, is higher in the darker‐colored skin of *Cyprinus carpio haematopterus* (yellow carp) and lower in the lighter, reddish‐hued skin of *Cyprinus carpio* var. *xingguonensis* (Xingguo red carp). Jiang's work demonstrated a clear association between GNQA and body color formation in both these carp varieties. Specifically, in *Cyprinus carpio* var. *xingguonensis* with its reddish body color and *Cyprinus carpio haematopterus* having a darker body color, the differential expression of *GNQA* was evident, further highlighting its role in determining the distinct body colors of these two types of carp (Jiang et al. 2014). Li et al. found that, in comparison to the surface‐type *Sinocyclocheilus*, the *ADCY9* (adenylate cyclase 9) and *GNQA* genes in the melanin synthesis pathway were down‐regulated in cave‐type *Sinocyclocheilus* compared to surface‐type golden barb. Additionally, the number of pigment cells in the skin of cave‐type *Sinocyclocheilus* was lower than in surface‐type golden barb. This suggests that the down‐regulation of *ADCY9* and *GNQA* affects pigment cell expression in the skin of surface‐type *Sinocyclocheilus*. The number of pigment cells in the skin of cave‐type *Sinocyclocheilus* fish was fewer than that in surface‐type golden barb, suggesting that the down‐regulated expression of *ADCY9* and *GNQA* ultimately affects melanocyte synthesis. In this study, KEGG functional enrichment analysis identified melanin synthesis genes (e.g., *ADCY9*, *GNQA*) in the brain tissues of both the *T. shilinensis* and the *T. xiangshuingensis*. These genes were down‐regulated in expression in brain tissues of the cave‐type *T. shilinensis* (Li et al. 2020). The fading of body coloration observed in the cave‐type *T. shilinensis* in this study is linked to the down‐regulated expression of *ADCY* and *GNQA* genes. Consequently, the melanin synthesized by melanocytes in the skin of the cave‐type *T. shilinensis* is likely reduced compared to that of the surface‐type *T. xiangshuingensis*, resulting in the semi‐translucent appearance of the *T. shilinensis* body. Further functional validation studies are necessary to ascertain whether these genes indeed affect the degradation of body color in the cave‐type *T. shilinensis* .

This does not imply the existence of color differences within the brain tissue itself, but rather strongly suggests that the brain, acting as a central regulatory hub, plays a pivotal role in the adaptive evolution of body color degeneration in cavefish by systemically coordinating this process. This perspective is firmly supported by research in fish neuroendocrinology. Substantial evidence indicates that changes in body coloration in fish are primarily regulated by the central nervous system. Studies have shown that neurons located in the hypothalamus synthesize and secrete Melanin‐Concentrating Hormone (MCH), which is transported via axons to the pituitary gland, released into the bloodstream, and subsequently acts remotely on melanocytes in the skin, directing the aggregation of pigment and thereby lightening body color (Kumbar and Ganesh 2022). The observed downregulation of *ADCY9* (adenylate cyclase 9) in this study is a key component of the cAMP signaling pathway, a canonical downstream pathway through which MCH and other neuropeptides exert their effects. Consequently, alterations in the expression of genes within such pigmentation‐related signaling pathways in the brain could potentially modulate systemic body pigmentation by influencing the activity of these neuroendocrine axes.

### 5.4 Down‐Regulation of Genes Involved in Vision‐Related Genes in *T. shilinensis*

One of the most striking phenotypes in cave fishes is the pronounced degeneration or complete absence of eyes. This visual system in fish is metabolically demanding, and the reduction or loss of this system in cavefish is an adaptation to conserve energy resources (Niven 2008). Research on the cavefish *A. mexicanus* has revealed a close association between the process of eye degeneration and the retinal pigment epithelium (RPE) (Ma et al. 2020). In fish, the initiation of vision in optic rod and cone cells occurs through photoisomerization. Specifically, within the visual chromatophore, namely the retina, the chromophore undergoes a transformation from the 11‐cis conformation to the all‐trans conformation. This process activates visual pigments, thereby initiating a phototransduction cascade. Ultimately, this cascade culminates in the generation of a response to light (Parker and Crouch 2010). Cyclic nucleotide‐gated (*CNG*) channels assume a pivotal role as mediators in the photoreceptor transduction pathway of optic rod and cone cells. Specifically, *CNGB1* (cyclic nucleotide‐gated channel beta 1) is classified among the dark vision genes. Mice with chronic deficiencies in this gene exhibit gradual degeneration, ultimately leading to the complete loss of optic rod and cone cells. Additionally, *CNGB1* has been found to have close associations with olfaction (Hüttl et al. 2005). In our study, *CNGB1* in the phototransduction pathway of the cave‐type *T. shilinensis* was down‐regulated compared to the surface‐type *T. xiangshuingensis*. These findings indicate this gene plays a critical role in the eye degeneration in the cave‐type *T. shilinensis* . *RDH8*, *LRAT*, and *CNGB1* are genes implicated in visual development. Retinol, essential for normal visual function, is converted by optic rod cells. In this process, all‐trans retinaldehyde is transformed into retinol through the catalytic activity of *RDH8* (retinol dehydrogenase 8). *RDH8* deficiency impedes M‐cones dark adaptation. This dark process is propelled by both the inner retinal visual cycle and the retinal pigment epithelium (*RPE*) visual cycle. Kolesnikov found that *RDH8* deficient mice exhibited normal M‐cone morphology but diminished visual acuity and reduced light response amplitude (Kolesnikov et al. 2015). Lecithin retinol acyltransferase (*LRAT*) catalyzes the synthesis of retinyl esters from fatty acids. It is specifically expressed in the retinal pigment epithelium (*RPE*) of the eye. The absence of *LRAT* leads to the loss of visual pigments in the *RPE* and degeneration of photoreceptors. Mutations in the *LRAT* gene can cause early‐onset retinal dystrophy (*LCA*), characterized by reduced visual pigments and progressive retinal degeneration or severe blindness. Infants with *LCA* typically exhibit significant visual impairment, markedly reduced bright‐eye ERGs, and distinctive ocular features (Sears and Palczewski 2016). In this study, the expression of *RDH8*, *LRAT*, and *CNGB1* was detected in the brain tissues of both *T. shilinensis* and *T. xiangshuingensis*. However, these genes were down‐regulated in *T. shilinensis* . The ocular deterioration in *T. shilinensis* is linked to abnormal or low expression of retinal development‐related genes. *T. shilinensis* has inhabited lightless cave environments for an extended period. Through adaptive evolution, down‐regulation of visual development‐related genes led to its eye degeneration.

### 5.5 Genes Involved in Olfaction‐Related Genes in *T. shilinensis* Showed Up‐Regulated

The olfactory system in fish assumes a crucial function in regulating their behavior, environmental acclimations, and life cycles. It detects diverse odor cues that dictate behaviors including feeding, alarm reactions, migratory routes, homing instincts, kin recognition, and reproduction (Zhu et al. 2017). Hu et al. discovered that *OLFR* (olfactory receptor) facilitated olfactory signaling in *C. elegans* ; it responds to odor stimulation by activating *GNAL* (guanine nucleotide‐binding protein G, alpha subunit, olfactory type), which in turn regulates *ADCY3* (adenylate cyclase type 3). They also identified that *C. elegans* olfactory signaling relies on *OLFR* activation, triggered by odor stimulation. GNAL regulates *ADCY3*, which activates *CNGB1* by elevating cAMP levels. This cascade results in Na^+^ and Ca^2+^ influx ions into olfactory sensory cells, stimulating olfactory function. Conversely, increased cAMP activates PKA (protein kinase), which phosphorylates *OLFR*, inhibiting ADCY3. *CALM* inhibits *CNGB1* and activates CAMK2 (calcium/calmodulin‐dependent protein kinase 2), which further inhibits *ADCY3* via phosphorylation, contributing to olfactory function recovery and adaptation (Hu et al. 2019). Our study identified olfactory transducer genes, including *OLFR*, *ADCY3*, PKA, and *CAMK2*, within the brain tissues of *Triplophysa*. These genes play integral roles in the regulation of the olfactory system. Interestingly, compared to the surface‐type *T. xiangshuingensis*, these genes were significantly up‐regulated in the brain tissues of the cave‐type *T. shilinensis* in response to the complete eye degeneration and loss as an adaptation to the dark environment. It is hypothesized that the observed up‐regulation of olfactory transducer genes may be attributed to the complete degradation and loss of eyes in *T. shilinensis* . This compensatory olfactory enhancement of their sense results from natural selection, facilitating adaptation to the dark cave environment.

### 5.6 The Coevolution of *Triplophysa* and *Sinocyclocheilus*

Convergent evolution refers to the independent emergence of similar traits in different lineages (Storz 2016). A study on mitochondrial oxidative phosphorylation (OXPHOS) genes in South American Gymnotiformes and African Mormyriformes (weakly electric fishes) showed that electric fishes had significantly lower evolutionary constraints (purifying selection pressure) on OXPHOS genes—especially Complexes IV and V—than non‐electric fishes, indicating convergent changes in selection pressure. Moreover, positive selection was found in Complex I genes of both lineages, with sites concentrated in proton transport‐related subunits (e.g., *ND2*, *ND4*, *ND5*). This suggests OXPHOS complexes may adapt to electric organs' high energy demands by improving proton transport efficiency and undergoing structural changes (e.g., *ND2‐L326T* mutation in both lineages), indirectly linking convergent function/structure adaptation to convergent selection pressure changes (Elbassiouny et al. 2019).

High‐altitude environments are a powerful driver of adaptive evolution in local organisms. On the Qinghai‐Tibet Plateau, three independent fish groups—Tibetan Loaches, Schizothoracine fishes, and Glyptosternoid fishes—all well adapt to the harsh local environmental conditions, serving as typical cases of convergent evolution (Yang et al. 2021).

This study shows that the brains of surface‐type and cave‐type Tibetan loaches have different regulatory effects on insulin secretion and energy metabolism. Compared to surface‐type Tibetan loaches, genes related to insulin secretion and energy metabolism in the brains of cave‐type *T. shilinensis* , such as *GLUT1*, *IRS1*, *PRKCA/PKCα*, and *ACSL*, are significantly downregulated. Genes related to circadian rhythm and behavior regulation, such as *CRY*, *FBXL3*, *CLOCK*, and *NPY2R*, also show significantly downregulated expression in the brains of *T. shilinensis* . Genes related to visual development, such as *RDH8*, *LRAT*, and *CNGB1*, are significantly downregulated in the brains of cave‐type *T. shilinensis* .

Through comparative transcriptomic analysis of brain tissues between cave‐type and Epigean‐type *Sinocyclocheilus*, it was found that cave‐type *Sinocyclocheilus* shares the same regulatory mechanisms as Tibetan loaches in the above three aspects. Compared to surface‐type *Sinocyclocheilus malacopterus* , the brain of hypogean‐type *Sinocyclocheilus rhinocerous* significantly regulates circadian rhythm, behavior, and insulin secretion. Genes related to insulin secretion, circadian rhythm, and behavior such as *cdk16*, *mtpn*, *hdac5*, *ubr1*, *ophn1*, and *faah* show significantly downregulated expression in the brains of cave‐dwelling *Sinocyclocheilus rhinocerous* . The brain plays an important regulatory role in the development of retinas and lenses in both *Sinocyclocheilus* species. Genes related to normal visual development such as *cryz* and *cep290* show significantly downregulated expression in the brains of cave‐type *Sinocyclocheilus rhinocerous* . In addition to these genes, comparisons with genes regulating these processes in Tibetan loaches revealed that *Sinocyclocheilus* shares the same genes as Tibetan loaches in regulating these processes (Li et al. 2020). Therefore, it is preliminarily proposed that Tibetan loaches and *Sinocyclocheilus* may exhibit convergent evolution.

Previous studies have also shown that some species of *Sinocyclocheilus*, due to living in extreme cave environments, exhibit a series of adaptive troglomorphic traits, including eye degeneration, lightened body color, elongated barbels, reduced metabolic rate, etc. (Jeffery 2001; Soares and Niemiller 2013). Most species of the genus *Triplophysa* are distributed in the Qinghai‐Tibet Plateau–centered region and karst caves in China. To adapt to the unique karst environment, fishes of this genus have also evolved some cave characteristics, such as eye degeneration, loss of body pigmentation, scale loss, barbel development, and physiological (energy metabolism) changes, with reduced metabolic rate and energy consumption. These may be convergent traits in cave‐type *Sinocyclocheilus* and *Triplophysa* when facing similar special environments (cave environments) (Chen and Yang 2005; Culver and Pipan 2019; Moran et al. 2014).

### 5.7 The Evolutionary Driving Force of Cave Adaptation in the *T. shilinensis*

Studies have revealed a significant downregulation of vision‐related genes (e.g., *RDH8*, *LRAT*, *CNGB1*) in the cave‐dwelling blind plateau loach ( *T. shilinensis* ). The functional degradation of these genes may partially stem from neutral evolution. In the dark cave environment, visual function is no longer favored by natural selection, and the accumulation of deleterious mutations leads to reduced gene expression. Meanwhile, the upregulation of olfaction‐related genes (e.g., *OLFR*, *ADCY3*) is a result of adaptive evolution, where natural selection preserves advantageous mutations. This helps the fish perceive food and the environment through olfaction in darkness, conforming to the evolutionary model of “neutral mutation + adaptive selection” (Kimura 1968; Moran et al. 2014).

Food resources are scarce in cave environments, so cave fish need to optimize energy allocation. The downregulation of genes related to insulin secretion and energy metabolism (*GLUT1*, *IRS1*, *PRKCA/PKCα*) observed in the study embodies the energy allocation theory. The fish reduce energy consumption for visual system and circadian rhythm‐related functions and divert more energy to key survival functions such as olfactory perception and energy storage. This trade‐off in energy allocation is an evolutionary strategy for long‐term adaptation to the low‐energy cave environment, which is consistent with the generally observed trait of “low metabolic rate” in cave fish. The Mexican blind cavefish ( *Astyanax mexicanus* ) maintains blood glucose stability through an insulin resistance mechanism. Mutations in the insulin receptor gene (*INSR*) reduce the sensitivity of cells to insulin, allowing blood glucose to remain at a relatively high level for an extended period to cope with unpredictable food acquisition (Olsen et al. 2023). At the same time, the sustained high expression of its appetite‐regulating genes (e.g., *NPY*) enhances feeding motivation, forming an energy storage strategy characterized by “high appetite – low metabolism”.

In contrast, the sympatrically distributed cave‐dwelling *T. shilinensis* and surface‐dwelling *T. xiangshuingensis* in this study may share part of the exogenous food input. Transcriptomic data show that insulin secretion‐related genes (*IRS1*, *PRKCA/PKCα*) in cave loaches are significantly downregulated, rather than exhibiting mutations related to insulin resistance. As a key substrate in the insulin signaling pathway, the reduced expression of *IRS1* decreases glucose transport and utilization (Hayashi et al. 2021). Although this mechanism also reduces energy consumption, it differs from the “blood glucose maintenance” strategy of Mexican blind cavefish: cave‐dwelling plateau loaches adapt to food restriction by reducing energy metabolism rate rather than maintaining high blood glucose, making this strategy more suitable for environments with “low‐frequency, low‐total” stable food input.

Light is the most prominent heterogeneous factor in cave environments. The gradient difference from “faint scattered light” to “complete darkness” directly drives the differentiation in the degree of visual system degradation and circadian rhythm gene expression in cave fish. Some semi‐open caves (e.g., light‐transmitting karst caves) have faint light, and cave fish living there exhibit incomplete degradation of the visual system. Studies have found that although the expression of their vision‐related genes (e.g., *RH1*) is downregulated, it is not completely silenced, and the retinal structure retains some photoreceptor cells (Li et al. 2020). The circadian rhythm genes (*CLOCK*, *CRY*) of these fish still maintain basic expression, enabling them to adjust their activity cycles through faint light.

In contrast, the cave environment in this study is completely dark, leading to more thorough visual degradation in cave loaches: genes related to retinal development (*RDH8*, *LRAT*, *CNGB1*) are all significantly downregulated. *RDH8* is responsible for retinol conversion, and its deficiency prevents the normal renewal of photoreceptor cells (Kolesnikov et al. 2015); *CNGB1*, as a phototransduction channel protein, has reduced expression that blocks light signal transmission (Hüttl et al. 2005). At the same time, the expression of circadian rhythm genes (*CRY*, *FBXL3*) is almost completely inhibited, indicating that cave‐dwelling plateau loaches have abandoned light‐based rhythm regulation and instead trigger activities through olfactory signals.

### 5.8 Conservation Strategies for Cave‐Dwelling *Triplophys* Loaches Based on Core Adaptive Genes

In this study, among the 27,194 differentially expressed genes identified in *T. shilinensis* , 15,006 were downregulated, with significant enrichment in pathways related to insulin secretion (e.g., *GLUT1*, *IRS1*), circadian rhythm (e.g., *CRY*, *CLOCK*), and visual development (e.g., *RDH8*, *CNGB1*). This pattern of “selective downregulation” reflects an evolutionary trade‐off between energy conservation and adaptation: by reducing energy expenditure in non‐essential processes, resources are reallocated to enhance critical traits such as olfaction (evidenced by upregulation of *OLFR* and *ADCY3*). This provides the first gene‐level evidence in the brain supporting an energy allocation optimization strategy in cavefish.

Furthermore, *T. shilinensis* exhibits convergent expression patterns with cave‐adapted species of the genus *Sinocyclocheilus*, including downregulation of visual and circadian rhythm genes and upregulation of olfactory genes. This suggests the existence of a “universal molecular adaptation pathway” in cavefishes, offering new insights into convergent evolution in evolutionary biology.

The key adaptation genes validated in this study can serve as molecular monitoring indicators for the conservation of cave‐dwelling *Triplophysa*. For instance: *GLUT1*, a central glucose transporter in the brain, reflects the population's metabolic state. Monitoring its expression in wild *T. shilinensis* could help detect energy stress early, potentially caused by groundwater pollution or food scarcity. The *OLFR* gene family, essential for olfactory perception, is directly linked to adaptive capacity in cave environments. Assessing its genetic diversity may help evaluate long‐term population viability. *CNGB1*, a key visual development gene, can assist in assessing whether light conditions in cave habitats have been artificially disturbed.

Transcriptomic analysis revealed 20,260 uniquely expressed genes in *T. shilinensis* and 33,439 in surface‐adapted *T. xiangshuiensis*. These species‐specific genes represent the “genetic core” underlying ecological adaptation and are crucial for maintaining population genetic diversity. Conservation efforts should prioritize preserving the genetic distinctiveness of each ecotype. This includes preventing the introduction of surface‐type loaches into cave ecosystems to avoid genetic introgression and loss of adaptive alleles (e.g., *OLFR*, *ACSL*).

In addition, given their small population sizes, genetic archives should be established, and the frequency of unique genes monitored regularly to prevent diversity loss due to genetic drift. Integrating molecular findings with the biological traits of *Triplophysa*, targeted conservation strategies can be proposed: The significant downregulation of circadian rhythm genes (e.g., *CRY*, *CLOCK*) in *T. shilinensis* suggests reduced activity and dispersal capacity. Conservation should thus focus on maintaining groundwater habitat connectivity and minimizing habitat fragmentation caused by construction or cave exploration.

Given the downregulation of energy metabolism genes (e.g., *GLUT1*, *IRS1*), human activities near caves should be strictly regulated to reduce groundwater pollution and ensure stable availability of food resources (e.g., plankton), thereby preventing population decline due to energy shortages.

## 6 Conclusion

This study compared the brain transcriptomes of sympatric cave‐adapted ( *T. shilinensis* ) and surface‐adapted (*T. xiangshuigensis*) loaches, yielding 60.74 Gb of clean data and 101,725 unigenes. A total of 27,194 differentially expressed genes (DEGs) were identified, with 15,006 downregulated and 12,188 upregulated in the cave morph. Functional enrichment and validation results indicate that *T. shilinensis* achieves cave adaptation through selective gene expression regulation: downregulating genes associated with insulin secretion (*GLUT1*, *IRS1*), circadian rhythm (*CRY*, *CLOCK*), and visual development (*RDH8*, *CNGB1*) to reduce energy consumption, while upregulating olfactory signaling pathway genes (*OLFR*, *ADCY3*) to compensate for visual loss. Furthermore, the observed DEG patterns converge with those reported in cave‐adapted *Sinocyclocheilus*, suggesting the potential existence of a universal molecular adaptation pathway in cavefishes. Functional Validation of Core Adaptive Genes: While this study has identified candidate genes such as *GLUT1* (energy metabolism), *CNGB1* (visual degeneration), and *OLFR*, their precise functions in cave adaptation require further validation. Future work should employ gene editing techniques to generate knockout or overexpression models, coupled with behavioral assays (e.g., foraging efficiency in darkness, response to light stimuli) and physiological measurements (e.g., blood glucose levels, insulin secretion), to elucidate their specific roles.

The current sample size (5 individuals per ecotype) permits only a preliminary exploration of molecular mechanisms. Future studies should expand the sampling scope to include various *Triplophysa* cave populations across the karst regions of Yunnan, Guizhou, and Guangxi. Combining whole‐genome resequencing will allow analysis of population genetic structure, genetic differentiation, and selection pressures, clarifying the evolutionary rates of cave‐adaptation genes. Ecological niche modeling should also be applied to predict the impacts of climate change on cave population distributions and assess long‐term extinction risks.

As this study focused on the brain transcriptome, future research should integrate metabolomic, proteomic (analyzing differential protein expression), and epigenomic (e.g., DNA methylation, histone modification) data to construct gene‐protein‐metabolite regulatory networks. This approach will reveal the multi‐layered regulatory mechanisms underlying cave adaptation. Furthermore, comparative genomic analyses across distantly related cavefish lineages, such as *Triplophysa*, *Sinocyclocheilus*, and *Astyanax mexicanus* , could identify conserved genetic modules associated with troglomorphic evolution.

*Triplophysa* cavefishes likely engage in complex interactions with cave microbiota and other benthic organisms. Future investigations could utilize 16S rRNA sequencing to analyze microbial community structures in cave water and fish guts, combined with stable isotope analysis to define dietary preferences and ecological niches. This would help explore the impact of host–microbe interactions on energy metabolism. Concurrently, studying reproductive isolation mechanisms (e.g., differences in spawning timing, courtship behavior) between cave and surface *Triplophysa* ecotypes will provide insights into the initial stages of speciation.

## Author Contributions

**Chunqing Li:** data curation (equal), formal analysis (equal), funding acquisition (equal), investigation (equal), project administration (equal), resources (equal), software (equal), writing – original draft (equal), writing – review and editing (equal). **Longting Wu:** formal analysis (equal), investigation (equal), methodology (equal), software (equal), validation (equal), visualization (equal), writing – original draft (equal), writing – review and editing (equal). **Fang Hu:** formal analysis (equal), investigation (equal), methodology (equal), software (equal), validation (equal), visualization (equal), writing – original draft (equal). **Shanyuan Chen:** conceptualization (equal), funding acquisition (equal), project administration (equal), resources (equal), supervision (equal), writing – review and editing (equal). **Heng Xiao:** conceptualization (equal), project administration (equal), resources (equal), supervision (equal), writing – review and editing (equal).

## Funding

This study was partially supported by the National Natural Science Foundation of China (32260310), the Top Young Talents Program of Ten Thousand Plan of Yunnan Province (YNWR‐QNBJ‐2018‐024), and Yunnan University Graduate Research Innovation Fund Project (Grant Nos. KC‐242410385).

## Ethics Statement

The animal study was reviewed and approved by the Animal Ethics and Welfare Committee at Yunnan University.

## Conflicts of Interest

The authors declare no conflicts of interest.

## Data Availability

The datasets presented in this study can be found in an online repository. Repository name: China National Center for Bioinformation, Genome Sequence Archive (GSA). Accession number: CRA027778, Access link: https://ngdc.cncb.ac.cn/gsa/browse/CRA027778.
